# Effects of *Rosa damascena* on reproductive improvement, metabolic parameters, liver function and insulin-like growth factor-1 gene expression in estradiol valerate induced polycystic ovarian syndrome in Wistar rats

**DOI:** 10.1016/j.bj.2022.05.003

**Published:** 2022-05-21

**Authors:** Mahbanoo Farhadi-Azar, Mehrdad Ghahremani, Fatemeh Mahboobifard, Mahsa Noroozzadeh, Parichehreh Yaghmaei, Fahimeh Ramezani Tehrani

**Affiliations:** a Reproductive Endocrinology Research Center, Research Institute for Endocrine Sciences, Shahid Beheshti University of Medical Sciences, Tehran, Iran; b Education Program in Reproduction and Development, Department of Obstetrics and Gynecology, Monash University, Melbourne, VIC, Australia; c Department of Pharmacology, School of Medicine, Shahid Beheshti University of Medical Sciences, Tehran, Iran; d Department of Biology, Science and Research Branch, Islamic Azad University, Tehran, Iran

**Keywords:** Polycystic ovary syndrome, Estradiol valerate, *Rosa damascena*, Insulin-like growth factor-1 (IGF-1)

## Abstract

**Background:**

Polycystic ovary syndrome (PCOS) is one of the most common endocrine disorders in reproductive-age women. The present study aimed to evaluate the effects of *Rosa damascena* (RD) extract in estradiol valerate (EV) induced polycystic ovary syndrome rats.

**Methods:**

Adult female Wistar rats were divided into control (n = 12) and PCOS groups (n = 36). The PCOS model was induced using EV (4 mg/kg/day), which was confirmed in 6 rats in each control and PCOS group by observation of irregular estrous cycles in vaginal smears and ovarian multiple cystic. Then, the rest of the control group (n = 6) and PCOS rats (n = 30 in 5 divided groups) were treated orally for 28 days with metformin (MET) as a positive control (200 mg/kg/day) and RD extract (400, 800, and 1200 mg/kg/day, respectively). Body and ovary weights, biochemical and histological parameters, and expression of the IGF-1 gene were measured.

**Results:**

Compared to the PCOS group, metformin and higher doses of RD extract (800 and 1200 mg/kg/day) significantly reduced BW, HOMA-IR, FBS, FINS, TG, LDL, TT, E2, LH, TC, and liver enzymes, and increased HDL and FSH levels. In addition, ovarian weight and CFs decreased, and the findings showed an increment in PFs, CLs, PAFs, AFs, and GFs. IGF-1 gene expression levels were significantly decreased (*p* < 0.001).

**Conclusion:**

RD extract seems to have the potential therapeutic effect of alleviating PCOS complications, and IGF-1 signaling may be involved in the beneficial effects of RD on PCOS.

**At a glance commentary**

**Scientific background on the subject**

Polycystic Ovary Syndrome (PCOS) is one of the most common endocrinopathies in reproductive-aged women. PCOS is associated with reproductive and metabolic disorders. The positive effects of Rosa damascena (RD), as a medicinal plant, on some reproductive and metabolic diseases are known; however, there is no report on PCOS.

**What this study adds to the field**

The present study indicates that the RD extract has the potential therapeutic effect of alleviating PCOS complications. Insulin-like growth factor 1 (IGF-1) signaling may be involved in the beneficial effects of RD on PCOS. RD treatment may be considered a novel treatment strategy for PCOS.

## Introduction

Polycystic Ovary Syndrome (PCOS) is one of the most common endocrine disorders in women during reproductive age, representing a prevalence of 6–10% worldwide [1]. Genetic and environmental factors, hormonal imbalances, and epigenetic changes in fetal life can play an important role in the PCOS growth cycle [1]. The most important criteria for the diagnosis of PCOS include hyperandrogenism (clinical or/and biochemical), oligo/anovulation, and polycystic ovaries [2]. Besides reproductive disorders, PCOS is associated with metabolic abnormalities such as insulin resistance (IR), impaired glucose tolerance, dyslipidemia, and obesity, as well as low-grade chronic inflammation and oxidative stress, all of which may lead to an increased risk of cardiovascular diseases and type 2 diabetes (T2DM) [3].

In women with PCOS, the prevalence of IR and compensatory hyperinsulinemia is 50–70% [4], and it can reach 95% in overweight women [5]. Hyperinsulinemia may lead to abnormal androgen secretion, impaired folliculogenesis, and the disruption of the menstrual cycle, all of which are important features of PCOS [6]. Abnormal androgen secretion stimulates the pituitary gland to over-secrete gonadotropin-releasing hormone (GnRH) through hypothalamic neurons, which increases luteinizing hormone (LH) [7].

Hyperandrogenism has also been shown to cause excessive insulin release by pancreatic β-cells. In addition, hyperinsulinemia reduces hepatic production of insulin-like growth factor (IGF) [8]. IGF-1, which primarily is synthesized by the liver, has hypoglycemic activity and shares 48 percent of the amino acid sequence with insulin [9]. According to research, increased IGF-I levels in PCOS patients is associated with IR [10].

Despite the noticeable prevalence of PCOS among women and its long-term side effects on women's health, the mechanisms underlying the pathophysiology of this syndrome have not yet been fully clarified, and treatments for PCOS, up to now, have been symptomatic [11]. Some commonly used chemical drugs for the management of PCOS include estrogen-progestin oral contraceptives, clomiphene citrate, and metformin [12].

Metformin (MET) is an oral hypoglycemic drug that was first introduced to treat T2DM [12]. Numerous studies support the use of this drug in PCOS because it increases insulin sensitivity and pregnancy rate by improving the menstrual cycle and inducing ovulation, and restores typical hormonal characteristics [12,13]. In addition, it reduces body weight (BW) and improves metabolic properties [13]. However, long-term treatment with it may lead to gastrointestinal upset, diarrhea, and other side effects that adversely affect the patient's standard of living [12]. Therefore, developing alternative treatment strategies that are more effective and cost-effective than chemical drugs is essential for women with immediate polycystic ovary syndrome.

*Rosa damascena* (RD) has been reported to have many medicinal and therapeutic effects, including anti-cancer, anti-diabetic, anti-inflammatory, anti-hypertensive, antioxidative properties [14]. This plant also showed beneficial effects on reducing the severity of labor pains, improving premenstrual syndrome and menstrual bleeding, treating primary dysmenorrhea, endometriosis, cardiovascular function and sexual behavior disorder [14]. RD stimulates the central nervous system to eliminate depression and cause happiness and vitality [14]. Based on the evidence, RD affects the release of GnRH from the hypothalamus and, therefore secretion of gonadotropin hormones from the anterior pituitary gland [15]. RD also has shown beneficial effects on IR and controlling diabetes, decreasing total cholesterol (TC), low-density lipoproteins (LDL), and triglyceride (TG), reducing liver enzymes in (Nonalcoholic fatty liver disease) NAFLD and increasing high-density lipoprotein (HDL) [16].

Flavonoids and some other phenolics have been suggested as important constituents of medicinal plants responsible for their medicinal properties. It has been reported that RD is in the subset of plants with the highest amount of flavonoids [17]. Quercetin is one of the main constituents in flowers of Rose species and the marc of RD flowers. This polyphenol shows the equivalent amount in different fresh flower extracts of Rose species [18]. Moreover, gallic acid is reported to be the most abundant phenolic compound of the extracts of RD, determined by the HPLC method [19].

Accordingly, the present study aimed to investigate the effects of standardized extract of RD based on quercetin and gallic acid on body and ovarian weight, ovarian histology, IR, lipid profile, liver enzymes, sex hormone levels and IGF-1 expression on a rat model of PCOS induced by estradiol valerate (EV). These results improve our understanding of PCOS development and may provide a novel treatment strategy for PCOS.

## Material and methods

### Preparation of *R. damascena* hydroalcoholic extract

Freshly dried petals of RD were purchased from Tabriz (East Azerbaijan, Iran) and authenticated by Dr. Hossein Nazemiyeh (Department of Pharmacognosy, Faculty of Pharmacy, Tabriz University of Medical Sciences). A voucher specimen (no: Tbz-FPh4043) has been deposited in the herbarium of the faculty of pharmacy.

The maceration method was used to obtain the extract. A total of 2 kg of dried fresh petals were powdered dissolved in Ethanol/Distilled water (70:30 v/v). The solution was then placed in the laboratory at a temperature of 25 °C for 48 hours. After filtration, the extract was concentrated at 50 °C by a rotary evaporator (Heidolph Co., Germany) and dried in an oven (Memmert Co., Germany) at 40 °C to dispel the alcohol completely. The final quantity of dry extract of RD was 250 g (yield, 12.5% (w/w)), and then was kept in the dark vials at 4 °C until the time of the experiments.

### High-performance liquid chromatography (HPLC) analysis and extract standardization for gallic acid and quercetin

**Fig. 1:**
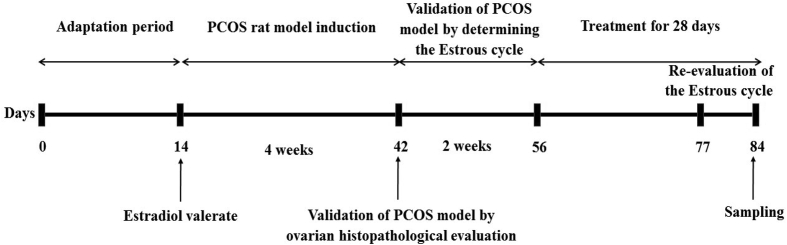
Schematic diagram of the experimental design. Estradiol valerate injection, PCOS induction, Confirmation of PCOS induction, treatment, and time of blood and tissues sampling.

Extract of RD was standardized for phenolic and flavonoid contents by HPLC, using gallic acid and quercetin (Sigma Aldrich Co., USA). The Details of HPLC analysis and comparison results of RD chromatogram with standard phenolic (gallic acid) and flavonoid (quercetin) chromatograms are presented in the Supplementary file (Fig. 1A and B). In RD samples, the concentrations of gallic acid and quercetin were 34.9 ± 3.2 mg/g dry extract and 6 ± 0.1 mg/g dry extract, respectively.

### Dose levels and dose selection

Referring to the Organization for Economic Co-Operation and Development (OECD) guidelines for acute toxicity, the LD50 of the extract of RD is >2000 BW [20,21]. According to Esfandiary et al. study, the oral LD50 of RD extract was determined to be >6 g/kg [22]. The oral dose range of ethanolic extract of RD used in studies is 50–1500 mg/kg [23]. Based on the abovementioned reports, the doses of 400, 800 and 1200 mg/kg were selected in this study.

We considered oral administration of RD based on the oral administration of RD in traditional medicine.

### Animals and ethics statement

In the present study, all experimental protocols were approved by the Ethics Committee of Islamic Azad University, Science and Research Branch, Tehran, Iran (Code: IR.IAU.SRB.REC.1397.175). Initially, forty-eight adult female Wistar rats (aged 10–12 weeks and weighing 180–220 g) were purchased from the Laboratory Animal Research Center (Royan Research Institute, Tehran, Iran). Before the experiment, the animals were adapted to the laboratory environment for 14 days. During the study, all rats were fed a standard diet (Pars Dam Company, Tehran, Iran) and water and kept in polypropylene cages (43 cm × 30 cm × 15 cm) under standard animal conditions (a diurnal cycle of 12 h light and 12 h dark from 07:00 AM, controlled room temperature 22 ± 3 °C, relative humidity 45–55%).

### Vaginal smear

Briefly, to determine the regularity or irregularity of the estrous cycle, a vaginal smear was collected in a rat vagina using a sterile plastic pipette tip containing 10 mL of normal saline (0.9% NaCl). Then one to two drops of this cell suspension were placed on a glass slide. After drying at room temperature, the slides were stained with Giemsa (Merck Co. Germany) and examined with a light microscope (OLYMPUS CX31 Co., Japan) at 100× magnification. Evaluation of estrus cycle stages was based on the ratio of three cell types: nucleated epithelial cells, corneal cells and leukocytes. In the estrous cycle, the prostate stage with the presence of nuclear epithelial cells (the first stage). The estrous stage with the presence of dominant corneal squamous epithelial cells (the second stage). In the Metestrus stage, there were nucleated epithelial cells, corneas, and leukocytes (the third stage), and in the distress stage, leukocytes were seen primarily (the fourth stage). To induce PCOS, only rats that were in the estrous stage of their reproductive cycle were selected [24].

### Induction of PCOS

A schematic diagram of the study design is shown in Fig. 1.

PCOS was induced in all experimental animals (n = 36) except the control group (n = 12) by a single intramuscular injection of EV (Aburaihan Co. Tehran, Iran) at a dose of 4 mg/kg/day (dissolved in 0.4 mL of sesame oil) for 28 days [25].

After 28 days, to ensure PCOS induction, vaginal smears were collected from each rat between 08:00 am and 9:00 am for 14 consecutive days to determine their estrous cycle.

Observation of irregular estrous cycles in vaginal smear (Rat with four disrupted estrus stages) by light microscopy was recognized as the main criterion for confirming the successful induction of the PCOS model. In addition, for complete validation of the model, 6 rats in each group were randomly selected, sacrificed by intraperitoneal (i.p.) injection of pentobarbital sodium (Sigma Aldrich Co. USA) (60 mg/kg) and their ovaries were evaluated for histopathology to confirm PCOS in rats. The presence of multiple cysts in the ovarian tissue indicates the main features of PCOS.

After modeling, the remaining animals in the control group (n = 6) and PCOS (n = 30 in 5 groups of 6 animals/distributed group) were used for the main future evaluations. Also, on day 77 (7 days before sampling), the reproductive cycle of each rat was re-evaluated.

### Study groups and experimental design

On day 56 of the experiment (after modeling), animals have divided randomly into five equal groups of six rats each:

**Control group:** healthy rats (Non-PCOS induced rats) that received a normal diet;
**PCOS group:** PCOS induced rats that received 1 ml/day normal saline.
**PCOS + MET (positive group):** PCOS rats that received MET (Merck Co., Germany) at a level of 200 mg/kg/day [26];
**PCOS + RD400:** PCOS rats received extract RD at the dose of 400 mg/kg/day;
**PCOS + RD800:** PCOS rats received extract RD at the dose of 800 mg/kg/day;
**PCOS + RD1200:** PCOS rats received extract RD at the dose of 1200 mg/kg/day.

All treatments had done by oral gavage for 28 consecutive days.

### Blood and tissue sampling

At the end of the 28-day treatment, the rats fasted for 12 h. In the estrus cycle of the estrus cycle, after deep anesthesia with an i.p. injection of 60 mg/kg pentobarbital sodium (Sigma Aldrich Co., USA), 5 mL of blood was obtained the abdominal aorta of all rats. It was centrifuged at 3000 rpm for 15 min (Kokusan H-11n Co. Japan). Isolated serum was stored at −20 °C until assayed for glycemic parameters, lipid profile, liver enzymes and sex hormone levels. After collecting blood and sacrificing the rats by heart incision, both ovaries were quickly removed, cleaned and weighed. The right ovaries were stored in Cryovials (Greiner Bio-One GmbH Co., Germany) for molecular tests and frozen in liquid nitrogen to freeze quickly. Finally, the collected tissues were stored at −80 °C. While the left ovaries were fixed in 10% formalin and histological analysis was performed. The BW was recorded weekly from the beginning to the end of the study.

### Biochemical analyses

The serum levels of biochemical parameters were assayed using an enzymatic colorimetric method, a biochemical autoanalyzer (BT-1500 Co., Italy), and a commercial diagnostic kit (Pars Azmoon Co., Iran). The commercial diagnostic kits category number was as follows; fasting blood sugar (FBS) (Cat no. 1-500-017); TG, (Cat no. 1-500-032); TC, (Cat no. 1-500-010); LDL (Cat no. 1-500-023) and HDL (Cat no. 1-500-012). An ELISA kit (Mercodia Co., Sweden, Cat no. 10-1247-07) was used to determine the serum levels of fasting insulin (FINS). HOMA-IR was calculated for each rat using the following equation: FINS (μU/ml) × FBS (mg/dl)/405 (>2.5 indicating a high index of IR) [27].

Serum concentrations for hormones levels were determined by ELISA. Serum TT (Total testosterone), LH and FSH (Follicle-stimulating hormone) were determined through an ELISA rat kits (Cusabio Biotech Co., China, Cat no. CSB-E05100r, CSB-E12654r and CSB-E06869r, respectively). Serum E_2_ was determined through an ELISA kit (Monobind Co., USA, Cat no. CA 92630). The endometrial variation coefficients for all hormones were < 10. Liver enzymes levels were measured by ELISA according to the manufacturer's instructions. ELISA kits were evaluated for ALP, AST and ALT (Cat no. 1-400-002, 1-400-018 and 1-400-019, respectively) and commercial diagnostic kits (Pars Azmoon Co., Iran).

### Histological analysis

For histological analysis, the ovaries were fixed in 10% formalin (Sigma Aldrich Co., USA) for three days at room temperature and then processed by a standard protocol and embedded in paraffin. The 6 μm thick ovaries were cut in series using a microtome (Leica RM2135 Co., Germany). All sections were placed on glass slides and stained using hematoxylin and eosin (H&E) (Merck Co., Germany). Ten representative sections were selected for each rat ovary to prevent the recurrence of follicles. The number of follicles, including Primordial follicles (PFs), preantral follicles (PAFs), antral follicles (AFs), graafian follicles (GFs), Corpora lutea (CLs), and cystic follicles (CFs), with The use of a microscope (Labomed Co., USA) equipped with a computer-connected camera, was evaluated and photographed.

### RT-qPCR analysis

Total RNA was extracted from ovarian tissue samples using RNeasy mini kit (Qiagen, Hilden, Germany, Cat no. 74104) according to the manufacturer's guidelines. The quantity and purity of RNA were measured using 1000 nanodrop (Thermo Scientific, Waltham, MA, USA) at 260 and 280 nm and then RNA was frozen at −80 °C. Then, for cDNA synthesis, the isolated RNA was transcribed using the Thermo-Fisher Scientific cDNA synthesis kit (Fermentas, USA, Cat no. K1621). The final reaction was performed in 20 μl volumes containing 10 μl SYBR Green PCR Master Mix Kit (Cat no. A190303, Amplicon Denmark), 1 μl CDNA, 0.5 μl forward primer, 0.5 μl reverse primer, and 8 μl nuclease-free water.

Were incubated for the time and temperature of the manufacturer. The IGF-1 and GAPDH (home maintenance gene) primer sequences were designed using the National Biotechnology Information Center (NCBI) website.

**IGF-1:** Forward: 5′- GTTGATAGGTGGTTGATGAATGG -3′.
 Reverse: 5′- AGAATGTAAAGAAAGGGCAGGG -3′.
**GAPDH:** Forward: 5′- AAGTTCAACGGCACAGTCAAGG -3′.
 Reverse: 5′- CATACTCAGCACCAGCATCACC -3′.

The starting sequences are listed in Table 1. RT-qPCR was performed in the ABI – step one system (Applied Biosystems Inc., Foster City, CA, USA). PCR conditions are as follows: 1 cycle, enzyme activation at 95 °C for 20 s, denaturation at 95 °C for 5 s, followed by annealing at 60 °C for 30 second, and prolongation at 72 °C for 30 second, then process repeated for 40 cycles. The relative amount of mRNA in each sample was calculated based on its threshold cycle (Ct) compared to Ct of GAPDH. Data were analyzed using 2^−ΔΔCT^ [28].

**Table 1:** The sequences of all primers.

Gene	Forward primer	Reverse primer
IGF-1	GTTGATAGGTGGTTGATGAATGG	AGAATGTAAAGAAAGGGCAGGG
GAPDH	AAGTTCAACGGCACAGTCAAGG	CATACTCAGCACCAGCATCACC

### Statistical analysis

The statistical analysis was performed using Graph Pad Prism® version 6.00 software (San Diego, CA, USA). Data were expressed as mean ± standard error of the mean (SEM), and comparisons of multiple samples were performed using one-way ANOVA and post-hoc Tukey test. *p* < 0.05 was considered statistically significant.

## Results

### Body and ovary weights

Table 2 shows that animal BW before PCOS induction was similar in all groups. Following a 28-day administration of EV, animal BW increased by at least 10% (*p* < 0.001) compared with the control group. MET therapy reversed this effect so that in the PCOS + MET group, this parameter decreased significantly by 14% compared to the PCOS group (*p* < 0.001). Similarly, treatment with 400, 800 and 1200 mg/kg of RD extract, significantly decreased this parameter by 7% (*p* < 0.01), 14% (*p* < 0.001) and 14% (*p* < 0.001), respectively, as compared with the PCOS group. The dose of 1200 mg/kg of RD extract significantly decreased animal BW by 7% more than 400 mg/kg, and there was no significant difference between the two doses of 800 and 1200 mg/kg in reducing BW [Table 3].

**Table 2:** Bodyweight before treatment in Control and PCOS groups.

Groups	BW before EV administration (g)	BW following a 28-day administration of EV (g)
Control	180.33 ± 3.08	185.50 ± 3.03∗∗∗
PCOS	180.50 ± 3.08	204.33 ± 1.43∗∗∗

Results are presented as mean ± SEM (Control, n = 12 and PCOS, n = 36).

∗∗∗*p* < 0.001 vs control group.

Abbreviations: PCOS: Polycystic ovary syndrome; BW: Body weight; EV: Estradiol valerate.

**Table 3:** Body and ovary weight after treatment in Control, PCOS, PCOS + MET, PCOS + RD400, PCOS + RD800, PCOS + RD1200 groups.

Groups	BW following a 28-day treatment (g)	Ovary weight (mg)
Control	196.50 ± 2.68	9.16 ± 0.72
PCOS	218 ± 1.93∗∗∗	16.16 ± 0.44∗∗∗
PCOS + MET	186.66 ± 2.07+++	10.33 ± 0.33+++
PCOS + RD400	201.50 ± 2.29++, $$, ##	15.16 ± 1.16∗∗∗
PCOS + RD800	186.16 ± 2.97+++	12.50 ± 0.28∗, +
PCOS + RD1200	185.66 ± 3.14+++	11.00 ± 0.57++

Results are presented as mean ± SEM (n = 6).

∗*p* < 0.05, ∗∗∗*p* < 0.001 vs control group.

+*p* < 0.05, ++*p* < 0.01 and +++*p* < 0.001 vs PCOS group.

$$*p* < 0.01 vs PCOS + RD800 group.

##*p* < 0.01 vs PCOS + RD1200 group.

Abbreviations: PCOS: Polycystic ovary syndrome; MET: Metformin; RD: *Rosa damascena*; BW: Body weight.

The ovary weight increased by 76% in the PCOS group compared with the control group [Table 3]. MET therapy reversed this effect so that in the PCOS + MET group, this parameter decreased significantly by 36% compared to the PCOS group (*p* < 0.001). Likewise, two doses of 800 and 1200 mg/kg of RD extract significantly decreased this parameter in comparison to the PCOS group by 22% (*p* < 0.05) and 31% (*p* < 0.01), respectively. Although treatment with 1200 mg/kg of RD extract significantly decreased animal ovary weight by 9% more than treatment with 800 mg/kg, the difference was not statistically significant compared to the PCOS group [Table 3].

### Glycemic parameters

**Fig. 2:**
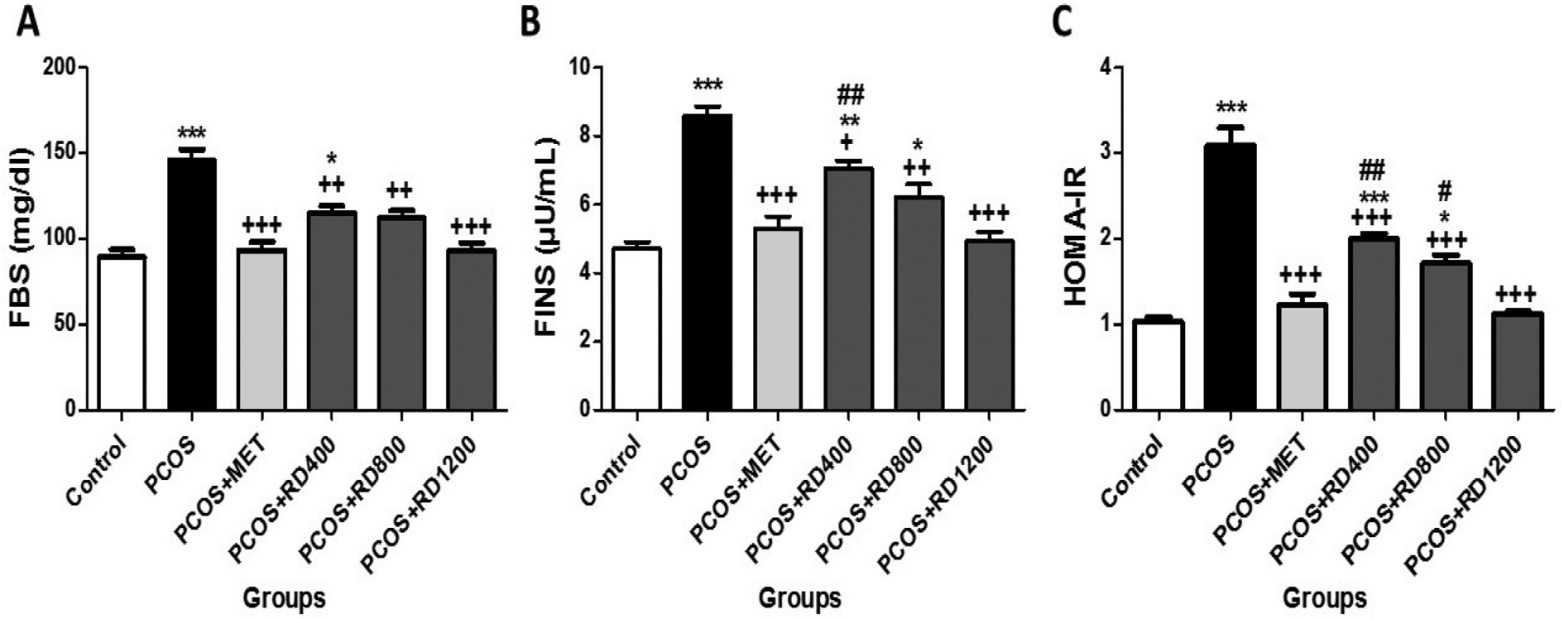
Evaluation of Serum glycemic parameters in Control, PCOS, PCOS + MTF, PCOS + RD400, PCOS + RD800, PCOS + RD1200 groups. Results are presented as mean ± SEM (n = 6). ∗*p* < 0.05, ∗∗*p* < 0.01 and ∗∗∗*p* < 0.001 vs control group. +*p* < 0.05, ++*p* < 0.01 and +++*p* < 0.001 vs PCOS group. #*p* < 0.05, ##*p* < 0.01 vs PCOS + RD1200 group. Abbreviations: PCOS: Polycystic ovary syndrome; MET: Metformin; RD: *Rosa damascena*; **(A)** FBS: Fasting Blood Sugar; **(B)** FINS: Fasting Insulin; **(C)** HOMA-IR: Homeostasis model assessment-Insulin resistance.

Fig. 2A shows that serum levels of FBS in the PCOS group increased significantly by 63% compared to the control group (*p* < 0.001). MET therapy reversed this effect so that in the PCOS + MET group, this parameter decreased significantly by 35% compared to the PCOS group (*p* < 0.001). Similarly, treatment with 400, 800 and 1200 mg/kg of RD extract, significantly decreased this parameter by 21% (*p* < 0.01), 23% (*p* < 0.01) and 36% (*p* < 0.001), respectively, as compared with the PCOS group. Although parallel to the increase of the doses of RD extract, the serum levels of FBS were decreased, the difference between RD extract treatment groups did not reach statistical significance.

Fig. 2B illustrates that serum levels of FINS in the PCOS group increased significantly by 82% compared to the control group (*p* < 0.001). MET therapy reversed this effect so that in the PCOS + MET group, this parameter decreased significantly by 38% compared to the PCOS group (*p* < 0.001). Similarly, treatment with 400, 800 and 1200 mg/kg of RD extract, significantly decreased this parameter by 17% (*p* < 0.05), 27% (*p* < 0.01) and 42% (*p* < 0.001), respectively, as compared with the PCOS group. Although compared to the PCOS group, treatment with 1200 mg/kg of RD extract decreased the serum levels of FINS by 15% more than treatment with 800 mg/kg of it; the difference did not reach significance.

Fig. 2C shows that levels of HOMA-IR in the PCOS group increased significantly by 200% compared to the control group (*p* < 0.001). MET therapy decreased this parameter so that in the PCOS + MET group, this parameter decreased significantly by 60% compared to the PCOS group (*p* < 0.001). Similarly, treatment with 400, 800 and 1200 mg/kg of RD extract, significantly decreased this parameter by 35% (*p* < 0.001), 44% (*p* < 0.001) and 63% (*p* < 0.001), respectively, as compared with the PCOS group. Although parallel to the increase of the doses of RD extract, the levels of HOMA-IR were decreased, the difference between RD extract treatment groups did reach statistical significance.

### Serum lipid profile

**Fig. 3:**
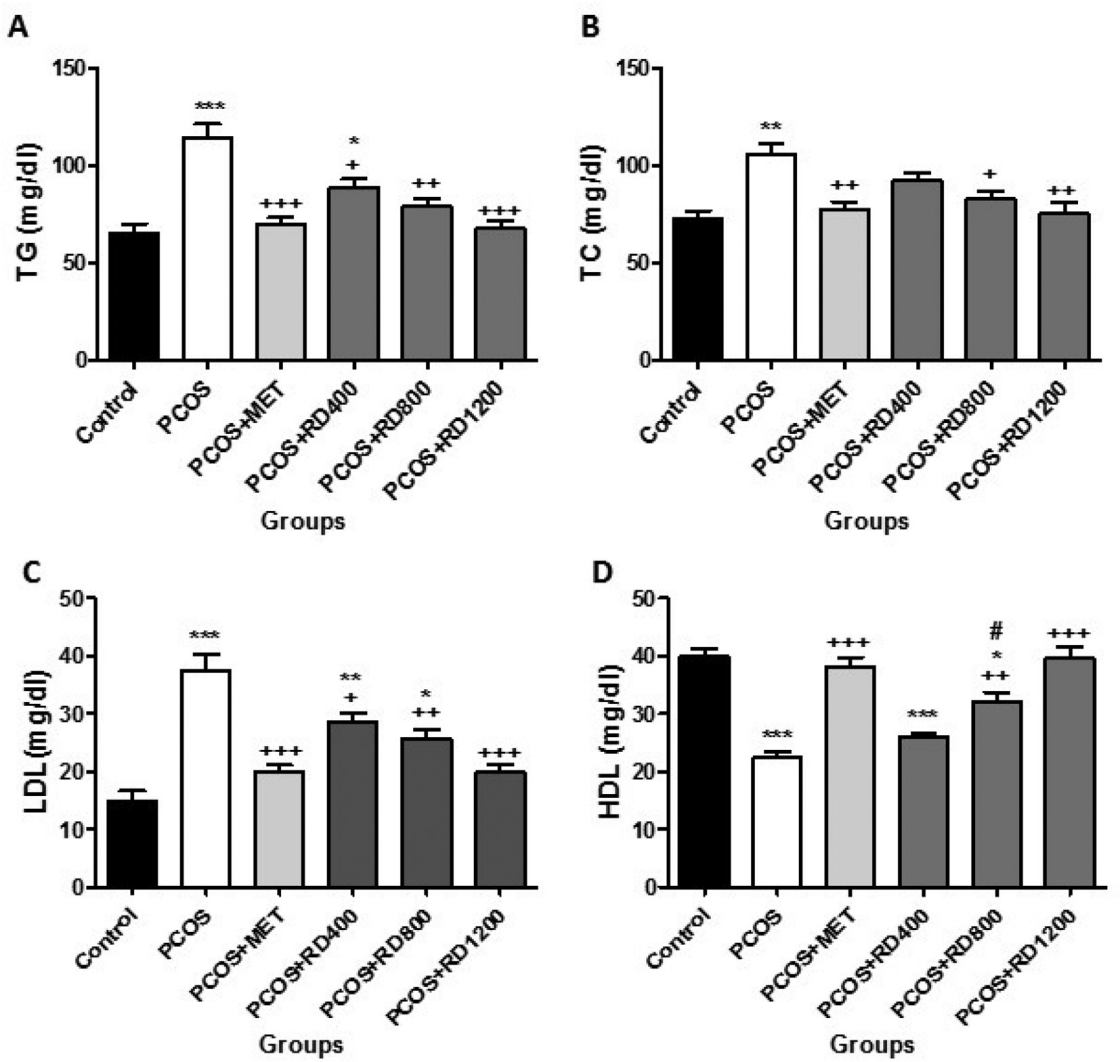
Evaluation of Serum lipid profile in Control, PCOS, PCOS + MET, PCOS + RD400, PCOS + RD800, PCOS + RD1200 groups. Results are presented as mean ± SEM (n = 6). ∗*p* < 0.05, ∗∗*p* < 0.01 and ∗∗∗*p* < 0.001 vs control group. +*p* < 0.05, ++*p* < 0.01 and +++*p* < 0.001 vs PCOS group. #*p* < 0.05 vs PCOS + RD1200 group. Abbreviations: PCOS: Polycystic ovary syndrome; MET: Metformin; RD: *Rosa damascena*; **(A)** TG: Triglyceride; **(B)** TC: Total Cholesterol; **(C)** LDL: Low-density lipoprotein; **(D)** HDL: high-density lipoprotein.

Fig. 3A shows that serum levels of TG in the PCOS group increased significantly by 76% compared to the control group (*p* < 0.001). MET therapy reversed this effect so that in the PCOS + MET group, this parameter decreased significantly by 51% compared to the PCOS group (*p* < 0.001). Similarly, treatment with 400, 800 and 1200 mg/kg of RD extract, significantly decreased this parameter by 38% (*p* < 0.05), 45% (*p* < 0.01) and 52% (*p* < 0.001), respectively, as compared with the PCOS group. Although parallel to the increase of the doses of RD extract, the serum levels of TG were decreased, the difference between RD extract treatment groups did not reach statistical significance.

Fig. 3B illustrates that serum levels of TC increased significantly by 44% compared to the control group (*p* < 0.01). MET therapy reversed this effect so that in the PCOS + MET group, this parameter decreased significantly by 26% compared to the PCOS group (*p* < 0.01). Likewise, two doses of 800 and 1200 mg/kg of RD extract significantly reduced this parameter by 21% (*p* < 0.05) and 28% (*p* < 0.01), respectively, when compared to the PCOS group, with no significant difference in reducing TC.

Fig. 3C shows that serum levels of LDL increased significantly by 150% compared to the control group (*p* < 0.001). MET therapy decreased this parameter by 46% as compared with the PCOS group (*p* < 0.001). Similarly, treatment with 400, 800 and 1200 mg/kg of RD extract, significantly decreased this parameter by 23% (*p* < 0.05), 31% (*p* < 0.01) and 47% (*p* < 0.001), respectively, as compared with the PCOS group. Although compared to the PCOS group, treatment with 1200 mg/kg of RD extract decreased the serum levels of LDL by 16% more than treatment with 800 mg/kg of it; the difference was not statistically significant.

Fig. 3D illustrates that serum levels of HDL decreased significantly by 44% compared to the control group (*p* < 0.001). MET therapy reversed this effect so that in the PCOS + MET group, this parameter increased significantly by 70% compared to the PCOS group (*p* < 0.001). Likewise, two doses of 800 and 1200 mg/kg of RD extract significantly increased this parameter in comparison to the PCOS group by 42% (*p* < 0.01) and 77% (*p* < 0.001), respectively, and the dose of 1200 mg/kg was more effective in enhancing HDL as compared with the dose of 800 mg/kg (*p* < 0.05).

### Serum liver enzymes

**Fig. 4:**
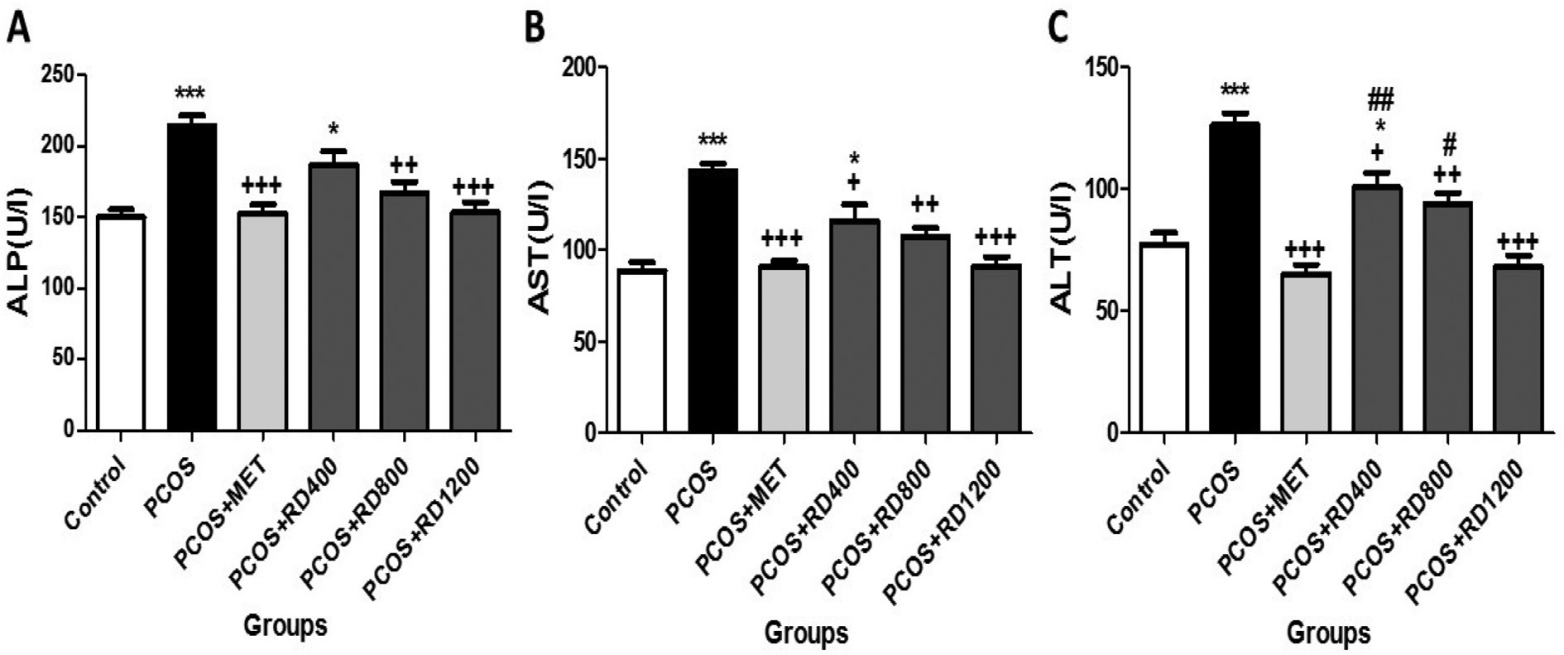
Serum levels of liver enzymes in Control, PCOS, PCOS + MET, PCOS + RD400, PCOS + RD800, PCOS + RD1200 groups. Results are presented as mean ± SEM (n = 6). ∗*p* < 0.05, ∗∗∗*p* < 0.001 vs control group. +*p* < 0.05, ++*p* < 0.01 and +++*p* < 0.001 vs PCOS group. #*p* < 0.05, ##*p* < 0.01 vs PCOS + RD1200 group. Abbreviations: PCOS: Polycystic ovary syndrome; MET: Metformin; RD: *Rosa damascena*; **(A)** ALP: Alkaline phosphatase; **(B)** AST: Aspartate aminotransferase; **(C)** ALT: Alanine aminotransferase.

Fig. 4A shows that serum levels of ALP increased significantly by 42% compared to the control group (*p* < 0.001). MET therapy reversed this effect so that in the PCOS + MET group, this parameter decreased significantly by 28% compared to the PCOS group (*p* < 0.001). Likewise, two doses of 800 and 1200 mg/kg of RD extract significantly decreased this parameter in comparison to the PCOS group by 22% (*p* < 0.01) and 28% (*p* < 0.001), respectively, as compared with the PCOS group. The difference between the two doses in increasing ALP was not statistically significant.

Fig. 4B illustrates that serum levels of AST increased significantly by 61% compared to the control group (*p* < 0.001). MET therapy reversed this effect so that in the PCOS + MET group, this parameter decreased significantly by 36% compared to the PCOS group (*p* < 0.001). Similarly, treatment with 400, 800 and 1200 mg/kg of RD extract, significantly decreased this parameter by 18% (*p* < 0.05), 25% (*p* < 0.01) and 36% (*p* < 0.001), respectively, as compared with the PCOS group. Although parallel to the increase of the doses of RD extract, the serum levels of AST were decreased, the difference between RD extract treatment groups did not reach statistical significance.

Fig. 4C shows that serum levels of ALT increased significantly by 63% compared to the control group (*p* < 0.001). MET therapy reversed this effect so that in the PCOS + MET group, this parameter decreased significantly by 48% compared to the PCOS group (*p* < 0.001). Similarly, treatment with 400, 800, and 1200 mg/kg of RD extract significantly decreased this parameter by 20% (*p* < 0.01), 25% (*p* < 0.01) and 46% (*p* < 0.001), respectively, as compared with the PCOS group, and the dose of 1200 mg/kg was more effective in reduced ALT than the dose of 800 mg/kg (*p* < 0.05).

### Serum hormonal levels

**Fig. 5:**
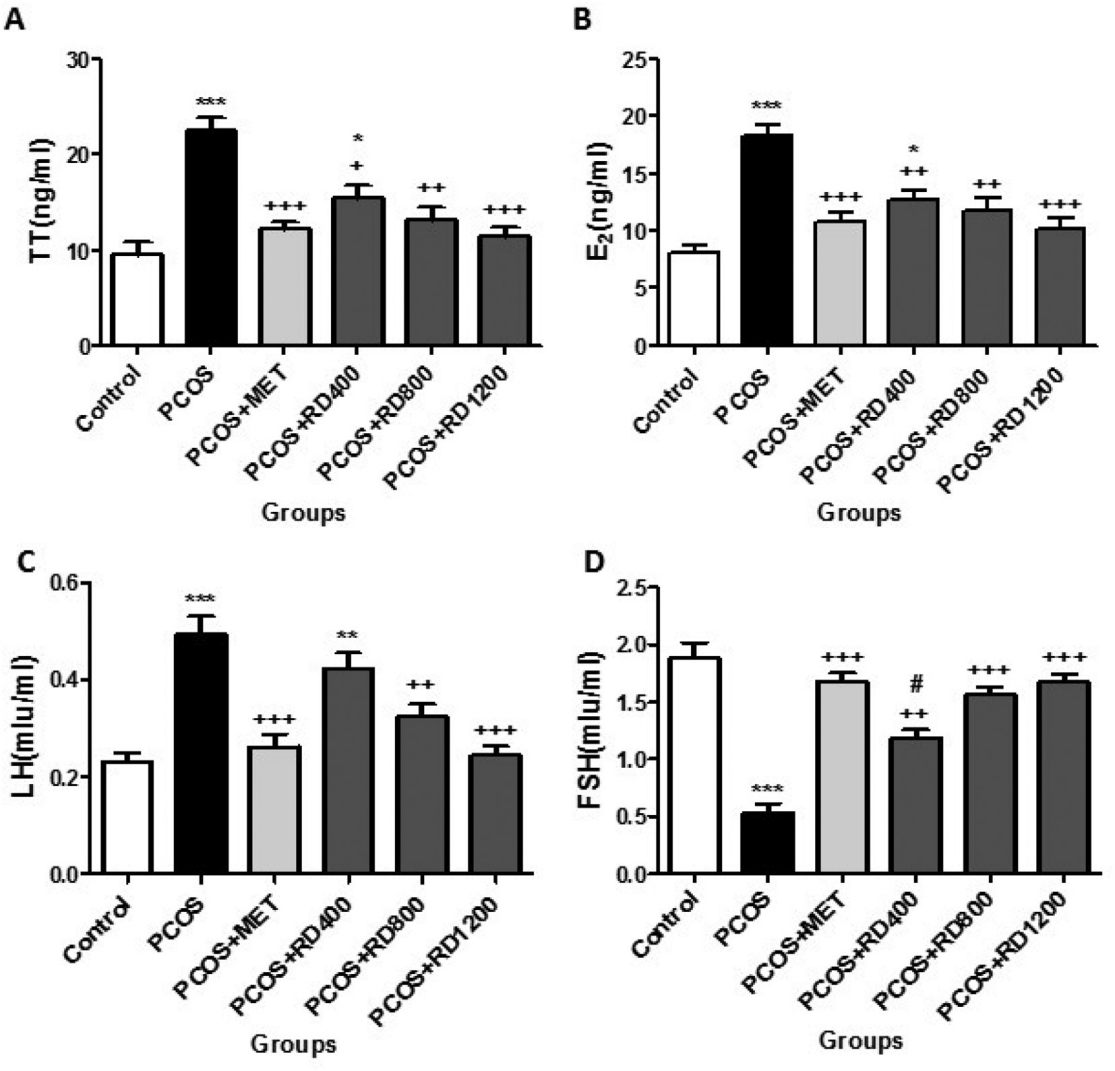
Comparison of Serum hormonal levels in Control, PCOS, PCOS + MET, PCOS + RD400, PCOS + RD800, PCOS + RD1200 groups. Results are presented as mean ± SEM (n = 6). ∗*p* < 0.05, ∗∗*p* < 0.01 and ∗∗∗*p* < 0.001 vs control group. +*p* < 0.05, ++*p* < 0.01 and +++*p* < 0.001 vs PCOS group. #*p* < 0.05 vs PCOS + RD1200 group. Abbreviations: PCOS: Polycystic ovary syndrome; MET: Metformin; RD: *Rosa damascena*; **(A)** TT: Total Testosterone hormone; **(B)** E_2_: Estradiol hormone; **(C)** LH: Luteinizing hormone; **(D)** FSH: Follicle-stimulating hormone.

Fig. 5A shows that serum levels of TT in the PCOS group increased significantly by 138% compared to the control group (*p* < 0.001). MET therapy reversed this effect so that in the PCOS + MET group, this parameter decreased significantly by 46% compared to the PCOS group (*p* < 0.001). Similarly, treatment with 400, 800 and 1200 mg/kg of RD extract, significantly decreased this parameter by 31% (*p* < 0.05), 41% (*p* < 0.01) and 48% (*p* < 0.001), respectively, as compared with the PCOS group. Although parallel to the increase of the doses of RD extract, the serum levels of TT were decreased, the difference between RD extract treatment groups did not reach statistical significance.

Fig. 5B illustrates that serum levels of E_2_ in the PCOS group increased significantly by 127% compared to the control group (*p* < 0.001). MET therapy reversed this effect so that in the PCOS + MET group, this parameter decreased significantly by 40% compared to the PCOS group (*p* < 0.001). Similarly, treatment with 400, 800 and 1200 mg/kg of RD extract, significantly decreased this parameter by 29% (*p* < 0.01), 35% (*p* < 0.01) and 44% (*p* < 0.001), respectively, as compared with the PCOS group. Although parallel to the increase of the doses of RD extract, the serum levels of E_2_ were decreased, the difference between RD extract treatment groups did not reach statistical significance.

Fig. 5C shows that serum levels of LH increased significantly by 113% compared to the control group (*p* < 0.001). MET therapy reversed this effect so that in the PCOS + MET group, this parameter decreased significantly by 46% compared to the PCOS group (*p* < 0.001). Likewise, two doses of 800 and 1200 mg/kg of RD extract significantly decreased this parameter in comparison to the PCOS group by 34% (*p* < 0.01) and 51% (*p* < 0.001), respectively. Although treatment with 1200 mg/kg of RD extract decreased the serum levels of LH by 17% more than treatment with 800 mg/kg of it, the difference was not statistically significant compared to the PCOS group.

Fig. 5D illustrates that serum levels of FSH decreased significantly by 71% compared to the control group (*p* < 0.001). MET therapy reversed this effect so that in the PCOS + MET group, this parameter increased significantly by 216% compared to the PCOS group (*p* < 0.001). Similarly, treatment with 400, 800 and 1200 mg/kg of RD extract, significantly increased this parameter by 122% (*p* < 0.01), 194% (*p* < 0.001) and 215% (*p* < 0.001), respectively, as compared with the PCOS group. The difference between two doses of 800 mg/kg and 1200 mg/kg of RD extract in enhancing FSH did not reach statistical significance.

### Ovarian histological findings

The ovarian histological results presented in Table 4 show that the number of PFs in the PCOS group decreased significantly by 60% compared to the control group (*p* < 0.001). By contrast, in the PCOS + MET group, the number of PFs increased by 118% compared to the PCOS group (*p* < 0.01). Likewise, two doses of 800 and 1200 mg/kg of RD extract significantly enhanced PFs in comparison to the PCOS group by 75% (*p* < 0.05) and 106% (*p* < 0.01), respectively. Although compared to the PCOS group, treatment with 1200 mg/kg of RD extract enhanced the number of PFs by 31% more than 800 mg/kg of it, the difference was not statistically significant.

**Table 4:** Number of follicles per ovary in Control, PCOS, PCOS + MET, PCOS + RD400, PCOS + RD800, PCOS + RD1200 groups.

Groups	PFs	PAFs	AFs	GFs	CLs	CFs
Control	26.66 ± 2.90	36.33 ± 3.28	26.66 ± 1.44	6.66 ± 0.34	14.33 ± 0.88	0
PCOS	10.66 ± 1.85 ∗∗∗	7.66 ± 1.45 ∗∗∗	6.33 ± 0.88 ∗∗∗	1.33 ± 0.29 ∗∗∗	7.66 ± 0.56 ∗∗∗	9.33 ± 0.66 ∗∗∗
PCOS + MET	23.33 ± 0.88++	32.00 ± 2.08+++	24.66 ± 1.45+++	6.33 ± 0.33+++	13.66 ± 0.65++	2.33 ± 0.32+++
PCOS + RD400	12.66 ± 0.66∗∗∗,#	23.66 ± 2.72∗,++	15.66 ± 1.20∗∗,++,#	3.66 ± 0.66∗∗,+	9.66 ± 0.78∗,#	6.00 ± 1.00∗∗∗,+,#
PCOS + RD800	18.66 ± 0.87∗, +	27.33 ± 2.84++	17.33 ± 1.45∗∗,++	4.33 ± 0.67∗,++	12.33 ± 0.86 +	5.33 ± 0.33 ∗∗∗,++
PCOS + RD1200	22.00 ± 1.52++	33.66 ± 2.18+++	23.00 ± 2.08+++	5.66 ± 0.32+++	14.33 ± 0.66+++	2.66 ± 0.88+++

Results are presented as mean ± SEM (n = 6).

∗*p* < 0.05, ∗∗*p* < 0.01 and ∗∗∗*p* < 0.001 vs control group.

+*p* < 0.05, ++*p* < 0.01 and +++*p* < 0.001 vs PCOS group.

#*p* < 0.05 vs PCOS + RD1200 group.

Abbreviations: PCOS: Polycystic ovary syndrome; MET: Metformin; RD: *Rosa damascena*; PFs: Primordial follicles; PAFs: Preantral follicles; AFs: Antral follicles; GFs: Graafian follicles; CLs: Corpora lutea; CFs: Cystic follicles.

The number of PAFs in the PCOS group reduced significantly by 78% compared to the control group (*p* < 0.001). MET therapy reversed this effect so that in the PCOS + MET group, the number of PAFs increased significantly by 317% compared to the PCOS group (*p* < 0.001). Similarly, treatment with 400, 800 and 1200 mg/kg of RD extract, significantly increased the number of PAFs by 208% (*p* < 0.01), 256% (*p* < 0.01) and 339% (*p* < 0.001), respectively, as compared with the PCOS group. Although parallel to the increase of the doses of RD extract, the number of PAFs was increased, the difference between RD extract treatment groups did not reach significance [Table 4].

The number of AFs in the PCOS group decreased significantly by 76% compared to the control group (*p* < 0.001). MET therapy reversed this effect so that in the PCOS + MET group, the number of AFs increased significantly by 289% compared to the PCOS group (*p* < 0.001). Similarly, treatment with 400, 800 and 1200 mg/kg of RD extract, significantly increased the number of AFs by 147% (*p* < 0.01), 173% (*p* < 0.01) and 263% (*p* < 0.001), respectively, as compared with the PCOS group.

Although compared to the PCOS group, treatment with 1200 mg/kg of RD extract enhanced the number of AFs by 90% more than treatment with 800 mg/kg of it; the difference did not reach significance [Table 4].

The number of GFs in the PCOS group reduced significantly by 80% compared to the control group (*p* < 0.001). MET therapy reversed this effect so that in the PCOS + MET group, the number of GFs increased significantly by 375% compared to the PCOS group (*p* < 0.001). Similarly, treatment with 400, 800 and 1200 mg/kg of RD extract significantly increased the number of GFs by 175% (*p* < 0.05), 225% (*p* < 0.01) and 325% (*p* < 0.001), respectively, as compared with the PCOS group. Although parallel to the increase of the doses of RD extract, the number of GFs was increased, the difference between RD extract treatment groups did not reach significance [Table 4].

The number of CLs decreased significantly in the PCOS group by 46% compared to the control group (*p* < 0.001). MET therapy reversed this effect so that in the PCOS + MET group, the number of CLs increased significantly by 78% compared to the PCOS group (*p* < 0.01). Likewise, two doses of 800 and 1200 mg/kg of RD extract significantly enhanced CLs in comparison to the PCOS group by 60% (*p* < 0.05) and 87% (*p* < 0.001), respectively. Although compared to the PCOS group, treatment with 1200 mg/kg of RD extract enhanced the number of CLs by 17% more than treatment with 800 mg/kg of it; the difference was not statistically significant [Table 4].

No CFs were observed in the control group. The number of CFs in the PCOS group was on average 9.33 ± 0.66 (*p* < 0.001). This number decreased significantly in the PCOS + MET group, compared to the PCOS group by 75% (*p* < 0.001). Similarly, treatment with 400, 800 and 1200 mg/kg of RD extract significantly decreased CFs in comparison to the PCOS group by 35% (*p* < 0.05), 42% (*p* < 0.01) and 71% (*p* < 0.001), respectively. Although compared to the PCOS group, treatment with 1200 mg/kg of RD extract decreased the number of CFs by 29% more than treatment with 800 mg/kg of it; the difference was not statistically significant [Table 4].

**Fig. 6:**
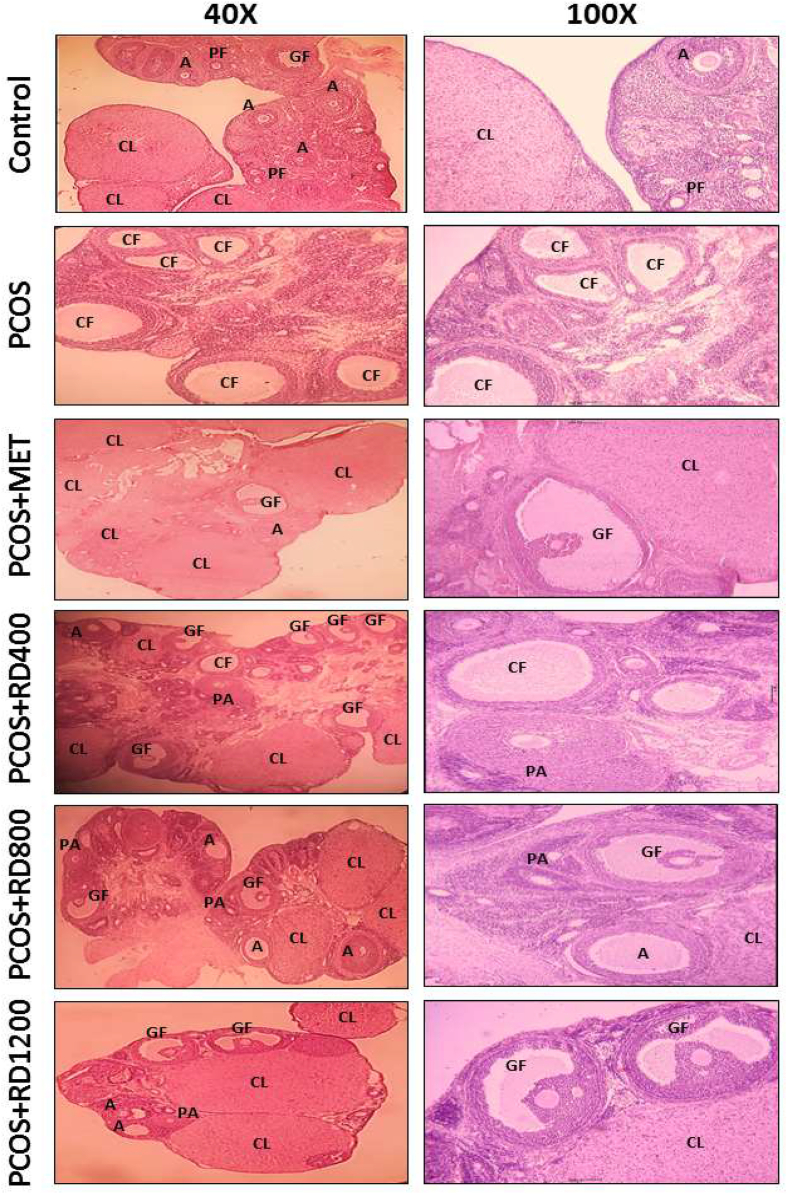
Photomicrographs of representative ovaries in Control, PCOS, PCOS + MET, PCOS + RD400, PCOS + RD800, PCOS + RD1200 groups. Hematoxylin-eosin (H&E) staining. Optical microscope (magnification 40× and 100×). Abbreviations: PCOS: Polycystic ovary syndrome; MET: Metformin; RD: *Rosa damascena*; PFs: Primordial follicles; PAFs: Preantral follicles; AFs: Antral follicles; GFs: Graafian follicles; CLs: Corpora lutea; CFs: Cystic follicles.

Fig. 6 illustrates photomicrographs of the ovaries of all study groups where PFs, PAFs, AFs, GFs, CLs, and CFs are identified. The control group exhibited normal ovarian architecture and normal follicular growth cycle with PFs, PAFs, AFs, GFs (as mature follicles), and CLs (as the indicator of ovulation), while ovarian sections of the PCOS group presented many CFs in their ovaries. Following treatment with MET (PCOS + MET) and RD (PCOS + RD400, PCOS + RD800, PCOS + RD1200), the effect of inducing PCOS in terms of disturbing normal follicular growth cycle was reversed so that the number of PFs, PAFs, AFs, GFs, and CLs took an upward trend as was comparable with the PCOS group.

### mRNA expression of IGF-1

**Fig. 7:**
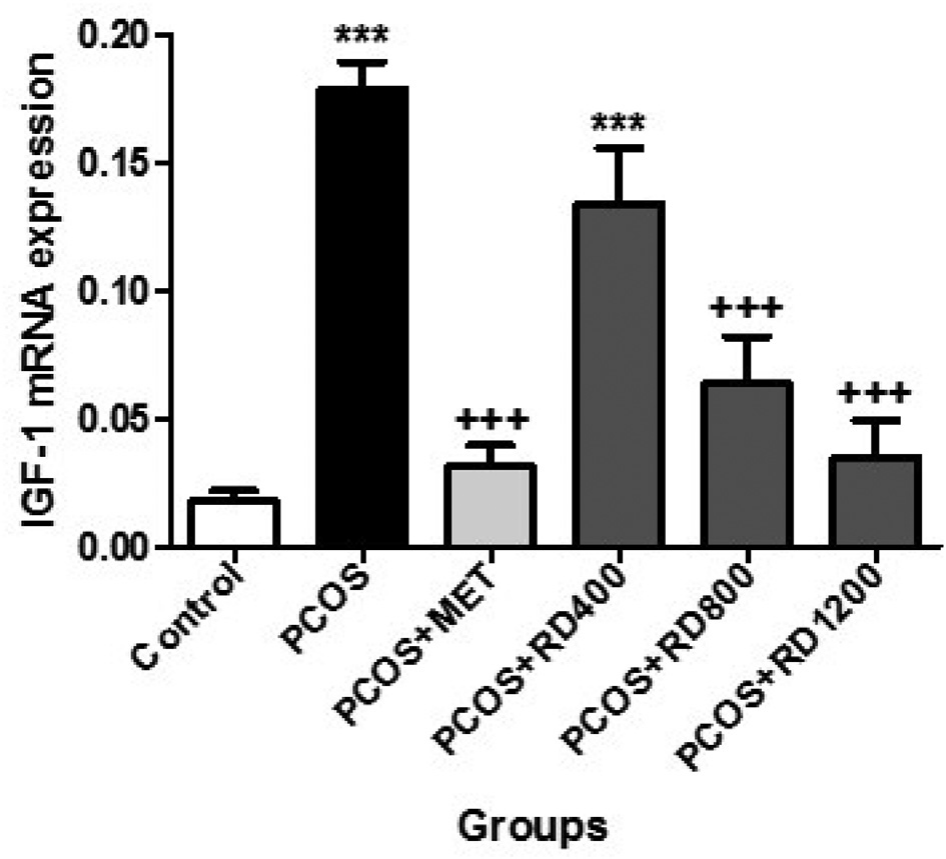
mRNA expression of IGF-1 in Control, PCOS, PCOS + MET, PCOS + RD400, PCOS + RD800, PCOS + RD1200 groups. Results are presented as mean ± SEM (n = 6). ∗∗∗*p* < 0.001 vs control group. +++*p* < 0.001 vs PCOS group. Abbreviations: PCOS: Polycystic ovary syndrome; MET: Metformin; RD: *Rosa damascena*; IGF-1: Insulin-like growth factor-1.

Fig. 7 shows that gene expression levels of IGF-1 in the PCOS group increased significantly by 952% compared to the control group (*p* < 0.001). By contrast, in the PCOS + MET group, this parameter decreased significantly by 82% compared to the PCOS group (*p* < 0.001). Likewise, two doses of 800 and 1200 mg/kg of RD extract significantly decreased gene expression levels in comparison to the PCOS group by 64% (*p* < 0.001) and 80% (*p* < 0.001), respectively. Although compared to the PCOS group, treatment with 1200 mg/kg of RD extract decreased the gene expression levels of IGF-1 by 16% more than treatment with 800 mg/kg of it; the difference was not statistically significant.

## Discussion

In the present study, we investigated the potential therapeutic effects of a standardized extract of RD based on quercetin and gallic acid on a rat model of PCOS induced by EV via investigating body and ovarian weight, ovarian histology in terms of the number of follicles and follicular growth cycle, glycemic parameters, serum lipid profile, liver enzymes, sex hormone levels, and IGF-1 expression. Although compared to the PCOS group, treatment with 400 mg/kg of RD extract was ineffective in improving some parameters, including reducing ovary weight, serum TC, HDL, ALP, LH, IGF-1 gene expression, and increasing PFs and CLs, higher doses of RD extract, including 800 and 1200 mg/kg, were found to significantly improve the status of all parameters. In terms of improving the levels of HOMA-IR, HDL, and ALT, the dose of 1200 mg/kg of RD extract was more effective than the dose of 800 mg/kg.

PCOS is the most common female endocrine disorder [1]. The disease leads to dysfunction of the reproductive mechanism and promotes several non-reproductive metabolic disorders such as IR, obesity, and hyperlipidemia [3].

Although PCOS is a common disease in women, its exact cause is still unknown. To study the cause of the disease and the effects of possible treatments, various methods have been used to induce PCOS in laboratory animals [29]. The use of drugs with estrogenic effects such as EV is a common method for inducing the PCOS model [30].

As expected, we found in the present study that EV efficiently induces PCOS in rats after 28 days of continuous use [25]. Hyperglycemia is a frequent feature of PCOS. In the present study, the levels of FINS and FBS in the PCOS group were significantly increased compared to the control group. This is consistent with the findings of Komal et al. and Ghafurniyan et al. [31,32]. MET is effective in improving hyperglycemia in patients with PCOS [33]. Similar to the findings of Wu et al. [34], we observed that MET-treated rats showed a significant reduction in FBS and FINS levels compared with the PCOS group. Our findings also showed that RD extract improved insulin sensitivity, which was evident by a decrease in FBG and FINS levels along with a significant decrease in IR. One possible hypothesis is that RD extract has an insulin-like effect and therefore lowers blood glucose levels [35]. Another possible mechanism may be related to the effects of RD extract on adiponectin concentration. Adiponectin increases insulin sensitivity by suppressing hepatic gluconeogenesis and stimulating muscle fatty acid oxidation [36].

Several factors, including insulin and thiazolidinediones, regulate adiponectin secretion [37]. Mohammadi et al. Showed that the concentration of adiponectin increases with the administration of RD extract [16]. In addition to the potential explanations for the effect of RD extract on improving glycemic parameters, quercetin, the main constituent of Rose species flowers, has been shown to promote pancreatic β-cells proliferation, glucose metabolism, and insulin secretion [38].

In the present study, the PCOS group showed a significant increase in liver enzyme levels and BW compared to the control group. These results were consistent with the findings of Pournaderi et al. [25]. In addition, the PCOS group showed a significant decrease in HDL levels but an increase in TG, TC, and LDL compared to the control group, which is consistent with the findings of Komal et al. [31]. Similar to our findings, Davoodi et al. found that RD extract reduced liver enzyme levels BW and improved the lipid profile in NAFLD induced by a high-fat diet (HFD) [39].

PCOS and NAFLD are considered to be the ovarian and hepatic manifestations of metabolic syndrome, respectively, and are both strongly associated with IR and obesity. PCOS women are more likely to develop NAFLD, and NAFLD, on the other hand, may increase the risk of PCOS [40]. This evidence might point to a connection between PCOS, NAFLD, IR, and obesity. IR causes the insulin receptor to malfunction, causing adipose tissue to fail to respond fully to the effect of insulin, resulting in an increase in fatty acid production and fat storage in the liver [41]. Increasing the synthesis of free fatty acids in the liver leads to increased levels of LDL and TG in the blood [42].

IGF-1, which primarily is synthesized by the liver, has hypoglycemic activity and shares 48 percent of the amino acid sequence with insulin [9]. According to research, increased IGF-I levels in PCOS patients is associated with IR [10]. IGF-1 is considered to be involved in regulating glucose and lipid metabolism and contributes to increasing insulin sensitivity and reducing the serum levels of triglycerides and free fatty acids [43]. Interestingly, in the present study, we found that, in addition to the impairment of glycemic parameters, serum lipid profiles, and liver enzymes in the PCOS group, IGF-1 was significantly increased, whereas this parameter was significantly decreased in the PCOS + MET group and PCOS groups treated with higher doses of RD extract, with improvement in glycemic and lipid parameters. To the authors' knowledge, there were no other studies on the effects of RD extract on IGF-1 gene expression and levels. Based on our findings, the authors speculate that in the crosstalk between PCOS, NAFLD, IR, and obesity, IGF1 might act as a linker and be a pathogenic factor for developing PCOS.

Mechanistically, IGF-1 is the main activator of Phosphatidylinositol-3-Kinase (PI3K), and PI3K/Akt signaling is one of the major pathways for insulin signaling; insulin induces the PI3K-Akt/protein kinase (PKB) pathway and mediates its regulatory effects on metabolism [44]. The Akt/PKB complex can transport glucose transporters, including Glucose transporter type 4 (GLUT4), to cell membranes, thereby increasing glucose uptake and lowering blood glucose levels [44]. On the contrary, mitogen-activated protein kinase (MAPK) activation has been linked to IR development by reducing GLUT4 expression and impairing glucose transport [45]. Chou et al. demonstrated that glucose levels are disrupted in AKT2 deficiency due to the inability of insulin to activate signaling pathways [46]. According to the Zhang et al. study, the therapeutic effect of berberine in reducing PCOS and IR symptoms in PCOS rats was associated with a mechanism involving increased GLUT4 regulation via PI3K/Akt activation and suppression of the MAPK pathway [47]. As a result, it is not unreasonable to assume that RD extract might improve IR and ovarian function via the IGF-1/PI3K/Akt pathway.

Elevated serum T and LH and low levels of E_2_ and FSH are the most important hormonal features for the evaluation of PCOS in women [48]. In this study, the PCOS group showed increased levels of LH, TT, and E2 but low FSH levels compared to the control group.

These findings were consistent with the results of Pournaderi et al. [25]. Elevated LH levels occur in PCOS due to disruption of the hypothalamic–pituitary axis, resulting in over-secretion of GnRH [7], which promotes the PI3K/Akt pathway, leading to overexpression of the ovarian CYP17A1 gene along with 17-α hydroxylase enzyme levels, increasing Progesterone to TT conversion [49]. Of note, in the current study, RD extract treatment reduced hyperandrogenism in the PCOS group, as observed by a significant reduction in TT and LH levels, leading to the improvement of the follicular growth cycle and ovulation stimulation. In parallel, it increased FSH levels. These findings might be attributed to the inhibitory effects of RD extract on the hypothalamic–pituitary axis, as reported by Jahromi et al. [15]. This potential effect of RD extract can be due to the presence of some plant compounds such as quercetin which counteract the biosynthetic mechanisms of LH-related androgens, leading to reduced TT and increased E_2_ levels [49].

In this study, EV caused ovarian dysfunction, which was confirmed by an increase in the number of CFs. This result which is in agreement with the findings of Pournaderi et al. [25], can be attributed to the high levels of LH hormone, leading to stimulating the ovaries to secrete androgens. Abnormal elevation in E_2_ levels is associated with the formation of CFs, leading to anovulation [50]. These harmful effects of EV on ovarian architecture were improved in response to treatment with PCOS + MET group and PCOS groups treated with higher doses of RD extract by the presence of increased mature follicles and CLs, indicating ovulation.

## Conclusions

In conclusion, our results in the present study on a rat model of PCOS revealed that although treatment with 400 mg/kg RD extract was ineffective in improving some negative features of PCOS, higher doses of extracts, including 800 and 1200 mg/kg, significantly improved all parameters. In terms of improving HOMA-IR, HDL, and ALT levels, the dose of 1200 mg/kg RD extract was more effective than the dose of 800 mg/kg. As a result, RD extract seems to have the potential therapeutic effect of alleviating PCOS complications. However, further research is required in the future.

## Funding

This study funded by the Research Institute for Endocrine Sciences, Shahid Beheshti University of Medical Sciences, Iran (grant number: 4-33147).

## Conflicts of interest

The authors declare that they have no competing interests.
