# Supplementary material for: Effects of Rosa damascena on reproductive improvement, metabolic parameters, liver function and insulin-like growth factor-1 gene expression in estradiol valerate induced polycystic ovarian syndrome in Wistar rats
Source: Biomed J. 2022 May 21;46(3):100538. doi: 10.1016/j.bj.2022.05.003 (PMC10209690; doi:10.1016/j.bj.2022.05.003)
Supplement: Supplementary file 1 [file mmc1.docx]

**SUPPLEMENTARY MATERIAL**

**HPLC analysis and chromatographic conditions**

The phenolic and flavonoid composition of RD extract was detected and quantified by HPLC, as described in the earlier research [1], although with some modifications. The system used was a Knauer-Azura ® HPLC system (Germany) with a P 6.1 L pump and a UV detector (DAD 2.1L). The detection wavelength was set to 280 nm. An Eclipse-XBD column (C18, ID 6 4.6 mm 250 mm, 5 m) was used for isolation. The injection volume was 20 μl, and the flow rate was 1 ml/min using the following solvent ratios for the mobile phase: H3PO4 0.3 g/l (pH 2.0) in water (solvent A) and acetonitrile (Merck Co., Germany) (solvent B). The slope wash was as follows: 0-1 min, from 60% A, 40% B; 1-20 min, from 60% A to 45% A, 40% B to 55% B; 20-21min, 45% A,55% B to 100% B; 21-25 min, from 100% B. Standard gallic acid (20 mg/ml, purity 99%) (CAS no. 5995-86-8) and quercetin (20 mg/ml, purity 95%) (CAS no. 117-39-5) were purchased from (Sigma Aldrich Co., USA) were dissolved separately in acetonitrile (HPLC grade). The RD extract sample was prepared as follows: 200 mg of the sample was dissolved in acetonitrile by HPLC grade and centrifuged for 10 minutes. A 0.45 m MS®PTFE syringe filter was used to filter the solution before it was injected into the HPLC apparatus. Calibration curves made separately with pure standards were used to quantify phenolic, and flavonoid compounds in RD extract and the amount of each compound in RD extract was expressed in mg/g dry extract.

**Results**

**Fig. 1A shows the HPLC chromatographic characteristics of a 280 nm RD extract, which shows two peaks among several others. Phenolics (gallic acid), peak 1 (RT: 3.30 min) and flavonoid composition (quercetin) peak 2 (RT: 12.27 min), comparison of RD chromatography with standard phenolic and flavonoid chromatography. Fig. 2B where Gallic acid shows a peak of 1 (RT: 3.47 minutes) and quercetin shows a peak of 2 (RT: 12.97 minutes).** **The retention time of RD extract was found to be consistent with standard compounds, indicating the presence of gallic acid and quercetin in RD extract. The concentration of gallic acid in the RD extract sample was calculated at 34.9±3.2 mg/g dry extract, and quercetin was 6±0.1 mg/g dry extract.**

**The determined values had a good linear relationship with the calibration curves of gallic acid and quercetin. Gallic acid and quercetin concentrations ranged from 10 to 40 μg/ml (Y = 16.87 x + 20.40, n = 3, R = 0.99) and 20 to 60**μ**g/ml (Y = 17.93 x - 86.65, n = 3, R = 0.98), respectively.**

**References**

[1] Kumar N, Bhandari P, Singh B, Gupta AP, Kaul VK. Reversed phase-HPLC for rapid determination of polyphenols in flowers of rose species. J Sep Sci 2008; 31:262-7.


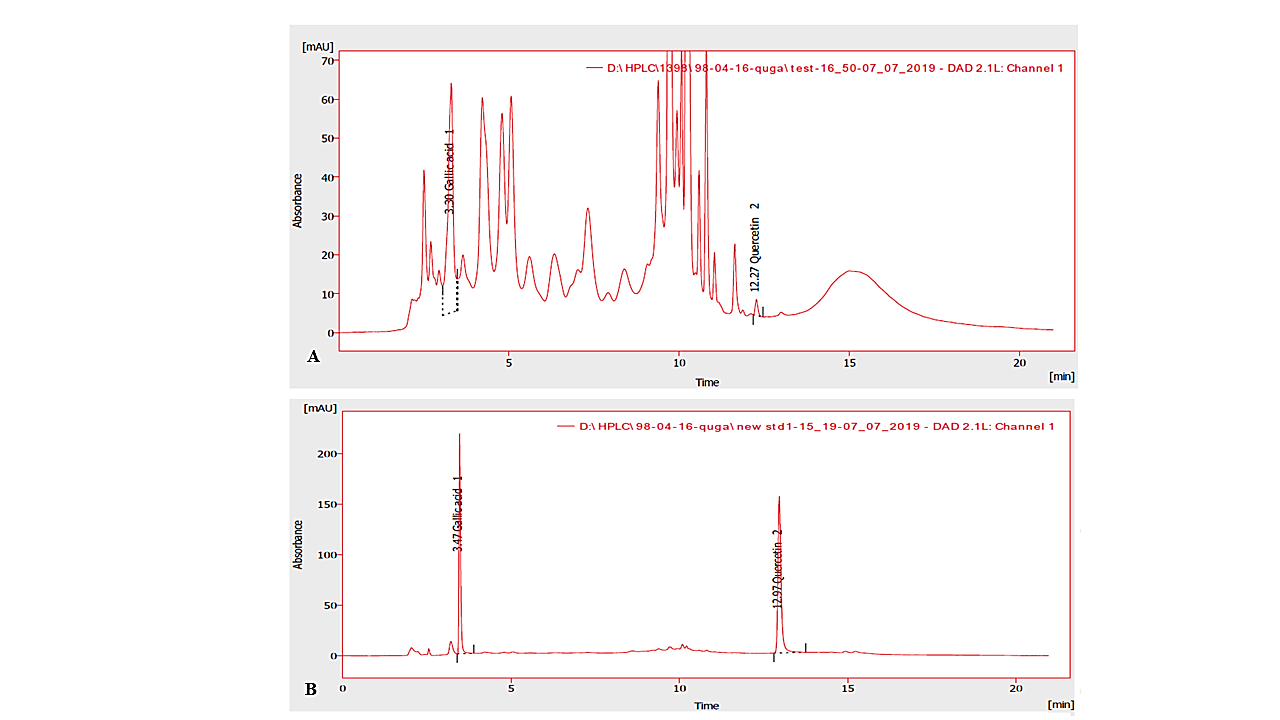


**Fig. 1(A)** HPLC-chromatogram of RD extract; **(B)** HPLC-Standard phenolic and flavonoid chromatograms.

Peak 1: gallic acid; Peak 2: quercetin.
